# Editorial - EJNMMI radiopharmacy and chemistry

**DOI:** 10.1186/s41181-016-0006-4

**Published:** 2016-03-21

**Authors:** Philip H. Elsinga

**Affiliations:** University of Groningen, Groningen, Netherlands

*EJNMMI Radiopharmacy and Chemistry* is a new journal within the EJNMMI family and is now ready to launch the first articles on-line. The aim of *EJNMMI Radiopharmacy and Chemistry* is to publish research in the field of new imaging and radionuclide based therapeutic agents that can be applied in nuclear medicine and molecular imaging.

This new journal provides an ideal platform for chemists, pharmacists and basic scientists to promote science, but also to exchange opinions and to provide updates on developments that have effect on availability of imaging agents for the clinic such as regulatory aspects.

*EJNMMI Radiopharmacy and Chemistry* reports original research articles, review papers, guidelines on application of imaging or radionuclide therapy agents, editorials, and letters to the editor. Research articles on novel radiochemistry, new radiopharmaceuticals including their first biological evaluation, molecular imaging agents including optical imaging, MRI, and hybrid probes, are the main focus of the journal. To translate imaging and radionuclide therapy agents to the clinic, legislative issues related to their production and safety can be presented as well in from of guidelines or position papers.

Radiopharmacy and radiochemistry are very essential for the development of nuclear medicine and molecular imaging and for its position in personalized medicine.

In this way, *EJNMMI Radiopharmacy and Chemistry* is very complementary to the other journals of the EJNMMI family, which focus mainly on (pre)clinical, technical and physical perspectives of nuclear medicine. With this new Journal we hope to establish a unique platform for our community.

Since the launch of *EJNMMI Radiopharmacy and Chemistry* in late October 2015 several manuscripts have been submitted, of which the first have now gone through the whole review process. So, we can now present the first number of accepted manuscripts. The Journal starts with a position paper developed by the EANM Radiopharmacy Committee on toxicity of radiopharmaceuticals, some new developments related to 68Ga-radiochemistry and an early preclinical studies. In addition some invited articles and original research articles are in the pipeline.

We are happy that a respectable number of scientist from all over the world have accepted the invitation to participate in the Editorial Board of *EJNMMI Radiopharmacy and Chemistry*. Their expertise covers a wide range of expertise in PET, SPECT and Molecular Imaging Radiopharmacy and Chemistry.

The members of the Editorial Board are: Mike Kilbourn (Ann Arbor, MI, USA), Jean Dasilva (Montreal, Canada), Jun Toyohara (Tokyo, Japan), Martin Behe (Villigen, Switzerland), Danielle Vugts (Amsterdam, The Netherlands), Clemens Decristoforo (Innsbruck, Austria), Ivan Penuelas (Pamplona, Spain), Bob Mach (Philadelphia, PN, USA), Klaus Kopka (Heidelberg, Germany), Jan Passchier (London, UK), Guy Bormans (Leuven, Belgium), Sally Schwarz (St. Louis, MO, USA), Sergio Todde (Monza, Italy), Ignasi Carrio (Barcelona, Spain), Angelika Bischof-Delaloye (Zürich, Switzerland), Ana Rey (Montevideo, Uruguay).

We hope you will enjoy reading the first articles in *EJNMMI Radiopharmacy and Chemistry*.

